# HIV Protease-Generated Casp8p41, When Bound and Inactivated by Bcl2, Is Degraded by the Proteasome

**DOI:** 10.1128/JVI.00037-18

**Published:** 2018-06-13

**Authors:** Sekar Natesampillai, Nathan W. Cummins, Zilin Nie, Rahul Sampath, Jason V. Baker, Keith Henry, Marilia Pinzone, Una O'Doherty, Eric C. Polley, Gary D. Bren, David J. Katzmann, Andrew D. Badley

**Affiliations:** a Division of Infectious Diseases, Mayo Clinic, Rochester, Minnesota, USA; b Division of Infectious Diseases, University of Minnesota, Minneapolis, Minnesota, USA; c HIV Program, Hennepin County Medical Center, Minneapolis, Minnesota, USA; d Department of Laboratory Medicine and Therapeutic Pathology, University of Pennsylvania, Philadelphia, Pennsylvania, USA; e Department of Biostatistics, Mayo Clinic, Rochester, Minnesota, USA; f Department of Biochemistry and Molecular Biology, Mayo Clinic, Rochester, Minnesota, USA; g Department of Molecular Medicine, Mayo Clinic, Rochester, Minnesota, USA; Emory University

**Keywords:** apoptosis, HIV, proteasome inhibitor

## Abstract

HIV protease is known to cause cell death, which is dependent upon cleavage of procaspase 8. HIV protease cleavage of procaspase 8 generates Casp8p41, which directly binds Bak with nanomolar affinity, causing Bak activation and consequent cell death. Casp8p41 can also bind Bcl2 with nanomolar affinity, in which case cell death is averted. Central memory CD4 T cells express high levels of Bcl2, possibly explaining why those cells do not die when they reactivate HIV. Here, we determine that the Casp8p41-Bcl2 complex is polyubiquitinated and degraded by the proteasome. Ixazomib, a proteasome inhibitor in clinical use, blocks this pathway, increasing the abundance of Casp8p41 and causing more cells to die in a Casp8p41-dependent manner.

**IMPORTANCE** The Casp8p41 pathway of cell death is unique to HIV-infected cells yet is blocked by Bcl2. Once bound by Bcl2, Casp8p41 is polyubiquitinated and degraded by the proteasome. Proteasome inhibition blocks degradation of Casp8p41, increasing Casp8p41 levels and causing more HIV-infected cells to die.

## INTRODUCTION

Understanding pathways of cell death that occur naturally in HIV-infected cells may instruct interventions that increase the proportion of HIV-infected cells that die. Although HIV infection kills most CD4 T cells that it infects, a small subset of HIV-infected cells survive, revert to a resting state, and become a latent reservoir for HIV. The major barrier to HIV eradication is the existence of this stable reservoir of latent virus, which resides predominantly in central memory CD4 T cells (1) and in follicular T helper cells (2). One proposed approach to eliminate these cells posits that reactivating HIV would allow immune recognition and, consequently, elimination of infected cells by intrinsic host effector immune mechanisms and/or due to the intrinsic cytotoxic properties of the HIV proteins themselves (i.e., the “shock-and-kill” hypothesis) (3). However, experimental testing *in vitro* (4) and *in vivo* has shown with remarkable consistency that reactivation from latency alone is insufficient to cause the death of the reactivating cell. For example, vorinostat treatment of antiretroviral therapy (ART)-suppressed HIV-infected patients caused reactivation of HIV but no reduction in the frequency of replication-competent HIV within resting CD4^+^ T cells (5). Therefore, the pathways of cell death that are activated by *de novo* HIV infection are seemingly not activated during reactivation from latency.

Multiple pathways have been described by which HIV-infected cells die as a consequence of HIV infection (reviewed in reference 6). One of these pathways is initiated by the intracellular expression of HIV protease, which, contrary to early reports, is catalytically active within the cytosol (7, 8). Expression of HIV protease alone in sufficient amounts is enough to kill some eukaryotic cells, and this phenomenon has been exploited to screen for inhibitors of HIV protease (9). The normal function of HIV protease is to cleave Gag-Pol to allow the initial steps of virus packaging. However, due to its degenerate substrate specificity, HIV protease also cleaves a number of host proteins (10–12). One host protein cleaved by HIV protease is procaspase 8 (13, 14); cells expressing a procaspase 8 mutant that is noncleavable by protease do not die following acute HIV infection *in vitro* (15). Conversely, certain drug resistance mutations in HIV protease impair its ability to cleave procaspase 8, decreasing Casp8p41 (see below) expression, and result in less CD4 T cell apoptosis than wild-type HIV protease (16).

HIV protease cleaves procaspase 8 between phenylalanines at positions 355 and 356, generating a 41-kDa fragment that we have named Casp8p41. Casp8p41 is seen only in HIV-infected cells (14), and Casp8p41 levels are predictive of future CD4^+^ T cell losses (16–18). Because Casp8p41 lacks the catalytic cysteine at position 360 of procaspase 8, it is catalytically inert, yet counterintuitively, it maintains the ability to induce cell death. Once generated, Casp8p41 translocates to the mitochondrion, where it adopts a BH3-like alpha-helical domain that binds to the BH3 groove of Bak, causing Bak activation and pore function that leads to loss of mitochondrial transmembrane potential, release of cytochrome *c*, and activation of downstream executioners of apoptosis (19–21). Because Casp8p41 is generated at a step in the HIV life cycle that occurs after integration, it follows that reactivation from latency should also generate Casp8p41. Indeed, our recent work, using cells from ART-suppressed HIV-infected patients, has shown that Casp8p41 is generated following HIV reactivation (22). Moreover, those studies showed that the cells in which latent HIV resides (e.g., central memory CD4 T cells) have an apoptosis-resistant phenotype and elevated expression of Bcl2. This led to the additional observations that Casp8p41 can also bind Bcl2 with nanomolar affinity and that, when Casp8p41 directly binds to Bcl2, the HIV-infected cell is not killed (22). Logically, therefore, inhibiting Casp8p41 binding to Bcl2 (thereby allowing Casp8p41 to bind Bak) enhances the number of HIV-infected cells that die (22, 23).

In this study, we tested the hypothesis that, after binding to Bcl2, the Casp8p41-Bcl2 complex is degraded and that blocking the degradation of Casp8p41 leads to increased Casp8p41 levels, greater proportions of HIV-infected cells dying, and a decrease in the number of HIV DNA-containing cells.

## RESULTS

### The Casp8p41-Bcl2 complex is polyubiquitinated.

**FIG 1:**
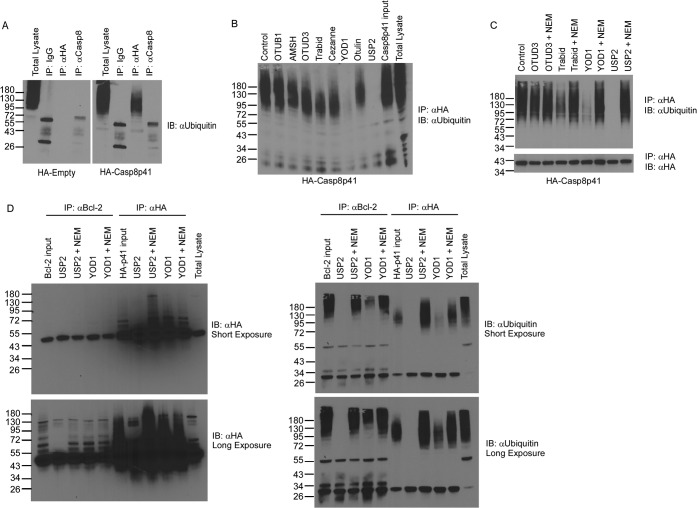
The Casp8p41-Bcl2 complex is ubiquitinated and targeted for proteasomal degradation. (A) 293T cells were transfected with HA-empty vector or HA-Casp8p41; immunoprecipitated (IP) with IgG control, anti-HA, or anti-caspase 8; and immunoblotted (IB) for ubiquitin. (B) Cytosolic extracts prepared as described for panel A were treated with various DUBs as indicated, immunoprecipitated with anti-HA, and immunoblotted for ubiquitin. (C) Cytosolic extracts prepared as described for panel A were treated with the DUBs OTUD3 and Trabid, the Lys48-specific YOD1, or USP2 in the presence or absence of the DUB inhibitor *N*-ethylmaleimide (NEM); immunoprecipitated with anti-HA; and immunoblotted for ubiquitin. (D) 293 T cells were cotransfected with Bcl-2 and HA-Casp8p41; treated with the DUB Trabid, YOD1, or USP2 in the presence or absence of the DUB inhibitor NEM; immunoprecipitated with anti-HA or anti-Bcl2; and then immunoblotted for HA (left) or ubiquitin (right), with short and long exposures depicted.

Regulated protein degradation is a prerequisite for cellular physiology, and the ubiquitin (Ub) proteasome system represents a major site of protein turnover. Reversible covalent modification of substrates with polyubiquitin chains drives their association with the proteasome and subsequent destruction. It has previously been documented that a complex of other proapoptotic proteins bound to their inhibitors is subject to proteasome-mediated degradation (e.g., proapoptotic NOXA bound by Mcl1 is degraded by the proteasome) (24). To test the idea that higher-molecular-weight forms of Ub-modified Casp8p41 exist and might also be degraded by the proteasome, immunoprecipitations were performed from cells expressing hemagglutinin (HA)-Casp8p41. Western blotting of the immunoprecipitated material using anti-ubiquitin antibody revealed the existence of higher-molecular-weight species within cells expressing HA-Casp8p41 (Fig. 1A). Furthermore, these higher-molecular-weight species were susceptible to the catalytic activities of a subset of deubiquitinating enzymes, specifically, USP2 and YOD1 (Fig. 1B and C). Whether ubiquitin-modified Casp8p41 was the result of Bcl2 binding was next addressed through coimmunoprecipitation and Western blotting using anti-HA and anti-ubiquitin antibodies. Immunoprecipitation of Bcl2 revealed association with HA-Casp8p41 (Fig. 1D, left), and ubiquitin-reactive species that were sensitive to DUB activity were detected regardless of whether Bcl2 or HA-Casp8p41 immunoprecipitation was first performed (Fig. 1D, right). Together, these data indicate that Casp8p41 can be polyubiquitinated. Furthermore, the observation that these species are sensitive to the enzymatic activities of YOD1 and USP2 suggests the ubiquitin chains are linked via lysine 48 (25–27). This suggests a model in which polyubiquitinated Casp8p41 in complex with Bcl2 is targeted to the proteasome for degradation.

### Proteasome inhibition increases Casp8p41 levels.

**FIG 2:**
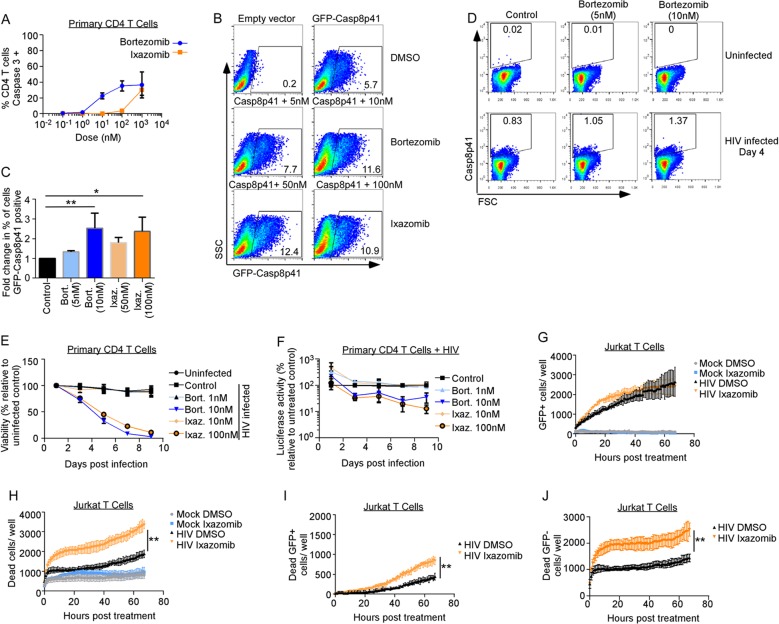
Proteasome inhibitors increase Casp8p41 levels and kill HIV-infected CD4 T cell cultures more than uninfected cultures. (A) Uninfected primary CD4^+^ T cells were treated with bortezomib or ixazomib at increasing concentrations for 48 h, and cell death was assessed by activated caspase 3 detection by intracellular flow cytometry. Depicted are the means and SD of the results of two experiments. (B and C) Jurkat CD4^+^ T cells were transfected with empty vector or GFP-Casp8p41 and then treated with control (DMSO), bortezomib, or ixazomib, and the percentage of cells that were GFP positive was analyzed 6 h later. (C) Mean (plus SD) data from three independent replicates of the experiment shown in panel B compared by a Kruskal-Wallis test. (D) Primary CD4 T cells were infected with HIV_IIIb_, treated with control or bortezomib, and assessed for intracellular Casp8p41 expression by flow cytometry. (E) Primary CD4 T cells were infected with HIV-Luc; treated with DMSO, bortezomib, or ixazomib; and monitored for viability by ATP content over time. (F) Primary CD4 T cells infected with HIV-Luc were treated with DMSO, bortezomib, or ixazomib and monitored for luciferase activity over time. (G and H) Jurkat T cells were mock or GFP-HIV infected, treated with DMSO or ixazomib, and monitored for GFP (G) or cell death (by cell permeability dye) (H) over time. (I and J) Cell death selectively measured in GFP-expressing cells (I) and GFP-negative cells (J) in panels G and H was monitored over time. *, *P* < 0.05; **, *P* < 0.01.

The clinical development of proteasome inhibitors (PIs) has altered the course of protein production diseases, such as multiple myeloma, in which plasma cells produce excessive amounts of monoclonal immunoglobulins. Long-term treatment with PIs over months to years causes semiselective death of myeloma cells (28) and improves the survival of affected patients (29). We tested whether the currently approved PIs bortezomib and ixazomib would block the degradation of Casp8p41. Dose-ranging toxicity studies of bortezomib or the active metabolite of ixazomib (MLN2238, here referred to as ixazomib) were performed in primary CD4 T cells. At doses at or below 10 nM and 100 nM, respectively, which are levels that are achievable in the sera of treated patients (30, 31), there was minimal nonspecific toxicity with bortezomib and less toxicity with ixazomib, as determined by active caspase 3 staining (Fig. 2A), consistent with the superior toxicity profile of ixazomib. Consequently, we used bortezomib at 5 and 10 nM and ixazomib at 50 and 100 nM in future experiments. Jurkat CD4^+^ T cells were transfected with green fluorescent protein (GFP)-tagged Casp8p41, treated with PIs, and analyzed for GFP-Casp8p41 positivity 6 h later; 10 nM bortezomib increased the percentage of cells positive for GFP-Casp8p41 by 2.5-fold relative to control cells (*P* = 0.009), and 100 nM ixazomib resulted in a 2.4-fold increase (*P* = 0.045) (Fig. 2B and C). This effect was confirmed in primary CD4 T cells infected *in vitro* with HIV_IIIb_, treated with bortezomib or control, and assessed for intracellular Casp8p41 positivity using a Casp8p41-specific monoclonal antibody (MAb) (Fig. 2D). Consistent with our previous reports (14, 17), Casp8p41 is present in HIV-infected T cells and not in uninfected cells. Furthermore, consistent with proteasome inhibitors increasing GFP-Casp8p41 in transfected cells (Fig. 2B and C), bortezomib treatment increased Casp8p41 expression in HIV-1-infected cells (Fig. 2D).

### Proteasome inhibitors kill HIV-infected CD4 T cells.

Because Casp8p41 is present only in HIV-infected cells (14, 17), increasing its expression might selectively cause the death of those HIV-infected cells as opposed to uninfected cells. This hypothesis was tested using 2 separate models of *in vitro* HIV infection. First, activated primary CD4 T cells were acutely infected with luciferase-expressing HIV and then treated with dimethyl sulfoxide (DMSO) control, bortezomib, or ixazomib. Bortezomib or ixazomib significantly increased killing of HIV-infected T cell cultures over time (*P* < 0.001 each) (Fig. 2E) at doses that did not alter the viability of uninfected primary CD4 T cells (Fig. 2A). Interestingly, the addition of bortezomib and ixazomib to the HIV-Luc-infected cells initially increased luciferase activity (see below for further discussion), but at later time points, the amount of luciferase activity decreased, consistent with HIV-infected cells dying (*P* = 0.023 and *P* = 0.008, respectively) (Fig. 2F). In our second model system, Jurkat T cells infected with GFP-HIV were treated with DMSO or ixazomib, and GFP expression was monitored over time (Fig. 2G). GFP-HIV-infected Jurkat cells treated with ixazomib had more cell death (*P* < 0.0001) than cells treated with control DMSO (Fig. 2H), but viability on mock-infected (uninfected) cells was not altered by ixazomib treatment (*P* = 0.72) (Fig. 2H). As not all cells in HIV-infected cultures are directly infected, we measured death specifically in the GFP-positive cells and found that ixazomib increased the number of dead GFP-positive cells compared to DMSO (*P* < 0.0001) (Fig. 2I). Conversely, ixazomib also increased cell death in GFP-negative HIV-exposed cells (*P* < 0.0001) (Fig. 2J). However, we have previously shown that GFP expression is lost during the process of apoptosis, so it is possible that some of the apparently GFP-negative cells in these cultures were indeed infected (22). Thus, in these experiments, PIs caused minimal toxicity in uninfected and unexposed T cells (Fig. 2A) yet enhanced killing of HIV-infected and, to a lesser extent, HIV-exposed cells.

### Proteasome inhibitors activate the HIV LTR predominantly via NF-κB.

**FIG 3:**
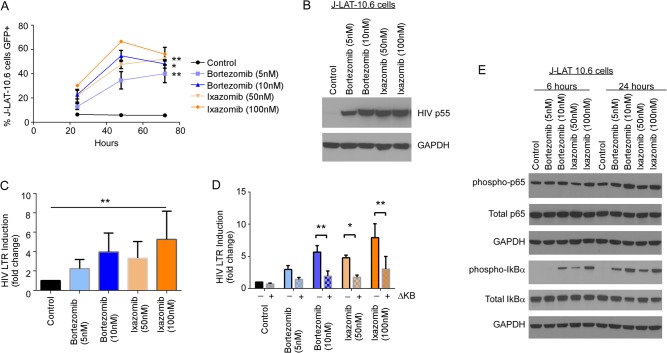
Proteasome inhibitors act directly on the HIV LTR to activate HIV in an NF-κB-dependent manner. (A) Chronically HIV-infected J-Lat 10.6 cells were treated with DMSO, bortezomib, or ixazomib and assessed for GFP expression over time. Depicted are means (±SD) of the results of four independent experiments. (B) J-Lat 10.6 cells treated as for panel A were assessed for HIV p55 Gag expression by Western blotting. (C) Jurkat CD4^+^ T cells transfected with an HIV-1 LTR firefly luciferase reporter construct were treated with control, bortezomib, or ixazomib, and luciferase activity was measured and normalized to that of Renilla luciferase. Depicted is fold luciferase induction relative to that of control-treated cells (means and SD of the results of five independent experiments). (D) Jurkat CD4^+^ T cells transfected with the HIV LTR luciferase construct with or without deletions in the NF-κB-binding site (ΔKb) were treated with control, bortezomib, or ixazomib and assessed for luciferase activity, normalized to that of Renilla luciferase. Depicted is fold luciferase induction relative to that of control-treated cells (means and SD of the results of two independent experiments). (E) J-Lat 10.6 cells were treated with control, bortezomib, or ixazomib for 6 or 24 h, as indicated, and expression of NF-κB p65, Ser536 phospho-NF-κB, IκBα, and Ser32 phospho-IκBα was assessed by Western blot analysis. *, *P* < 0.05; **, *P* < 0.01.

Above, we observed that ixazomib causes a transient increase in HIV Luc (Fig. 2E) and HIV GFP (Fig. 2F) expression in infected cultures. Since bortezomib has been previously shown to activate NF-κB (32) and since the HIV long terminal repeat (LTR) is driven by NF-κB, we questioned whether ixazomib might directly activate HIV replication through NF-κB. To begin, we used J-Lat 10.6 cells, which express GFP upon HIV reactivation, to assess if ixazomib directly impacts HIV replication. Treatment with ixazomib and with bortezomib led to an increase in GFP expression over time (Fig. 3A) that was coupled with HIV p55 Gag protein expression by Western blot analyses (Fig. 3B).

We next evaluated whether these effects on HIV replication were mediated by NF-κB transactivation of the HIV LTR. Luciferase reporter constructs in which either the HIV LTR was linked to luciferase (HIV-Luc) or the HIV LTR missing the NF-κB-binding sites was linked to luciferase (HIVΔκB-Luc) were transfected into Jurkat T cells, and the transfection efficacy was normalized to that of cotransfected Renilla, as previously described (33). Treatment with bortezomib or ixazomib resulted in a dose-dependent increase in luciferase expression (normalized to that of Renilla luciferase), demonstrating a direct effect on HIV LTR-mediated replication in cells containing HIV-Luc (Fig. 3C). Consistent with this effect being mediated predominantly through NF-κB transactivation of the HIV LTR, Luc activity was significantly less augmented in the cells containing the HIVΔκB-Luc reporter (Fig. 3D). Internally consistent with these findings, the negative repressor of NF-κB, IκB, was inactivated (through Ser32 phosphorylation [34]), and the p65 subunit of NF-κB was activated (through Ser536 phosphorylation) within 6 h of treating J-Lat 10.6 cells with bortezomib or ixazomib (Fig. 3E).

### Selective killing of HIV-infected cells by proteasome inhibitors depends upon Casp8p41.

**FIG 4:**
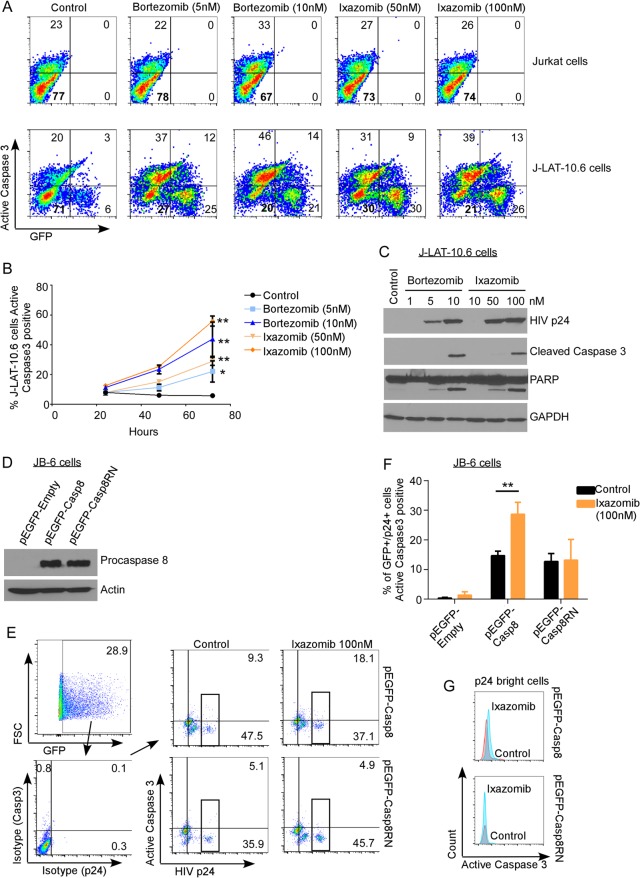
Proteasome inhibitors induce apoptosis in HIV-infected T cells, but not uninfected T cells, that depends upon procaspase 8, which is cleavable by HIV protease. (A) Chronically HIV-infected J-Lat 10.6 cells and uninfected parental Jurkat cells were treated with DMSO, bortezomib, or ixazomib and monitored for apoptosis by intracellular expression of active caspase 3. (B) Polled data depicting means (±SD) of the results of four independent experiments, as shown in panel A. (C) J-Lat 10.6 cells treated with DMSO, bortezomib, or ixazomib were analyzed by immunoblotting for HIV p24, cleaved caspase 3, cleaved PARP, and GAPDH. (D) Caspase 8-deficient JB-6 cells were transduced with VSV-G-pseudotyped HIV and then transfected with EGFP, EGFP-caspase 8, or EGFP-caspase8RN. Caspase 8 expression was confirmed by Western blotting (with a mutant of caspase 8 that is not cleaved by HIV protease and therefore does not generate Casp8p41). (E) HIV-infected JB-6 cells as in panel D were treated with ixazomib or control, and cell death was determined by active caspase 3 in caspase 8-expressing (GFP^+^) and HIV-infected (p24^+^) cells. (F) Pooled data from three replicates of the experiment shown in panel E showing mean and SD. (G) Active caspase 3 expression was also compared in the subset of HIV p24-bright cells (boxed in panel E). *, *P* < 0.05; **, *P* < 0.01.

**FIG 5:**
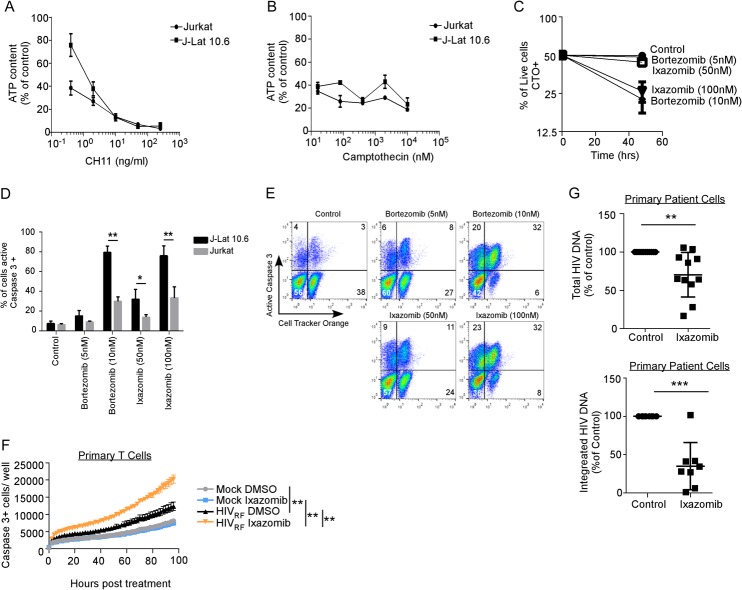
Mixtures of HIV-infected and uninfected T cells, treated with proteasome inhibitors, select for the preferential survival of uninfected cells. (A) Jurkat T cells and chronically HIV-infected J-Lat 10.6 cells were treated with apoptosis-inducing anti-Fas (CH11), and cell survival was monitored by intracellular ATP. Depicted are means (±SD) of the results of triplicate experiments. (B) Jurkat T cells and J-Lat 10.6 cells were treated with camptothecin, and cell survival was monitored by intracellular ATP content. Depicted are means (±SD) of the results of triplicate experiments. (C) J-Lat 10.6 cells were labeled with the lipophilic dye CTO to distinguish them from unlabeled Jurkat T cells, and the two cell types were mixed at a 1:1 ratio. T20 was added to prevent spreading HIV infection, and then the cells were treated with control, bortezomib, or ixazomib and analyzed for active caspase 3 staining. Depicted is the proportion of live (active caspase 3-negative) cells that were also CTO-positive (J-Lat 10.6) cells (means ± SD of the results of three independent experiments). (D) Pooled data from three replicates of the experiment shown in panel C depicting means and SD. (E) Representative flow data from panels C and D; cell death in the CTO-positive (J-Lat) and CTO-negative (Jurkat) cells can be seen in the same sample. (F) Primary activated CD4 T cells were mock infected or infected with HIV-1_RF_, treated with ixazomib (100 nM) or vehicle control, and assessed for active caspase 3 expression over time. (G) Freshly isolated CD4^+^ T cells from HIV-infected ART-suppressed patients were treated with ixazomib or control and ART (T20, EFV, or RAL) to prevent spreading infection. After 96 h, cells were harvested, both total (top) (*n* = 11 patients) and integrated (bottom) (*n* = 8 patients) HIV DNA copies per million cells were determined, and the values were compared to the control by one-sample *t* test. *, *P* < 0.05; **, *P* < 0.01; ***, *P* < 0.001.

Next, we assessed whether the ixazomib-induced death of HIV-infected cells was dependent upon the presence of Casp8p41. HIV-infected J-Lat 10.6 cells or parental Jurkat CD4 T cells were treated with vehicle control, bortezomib, or ixazomib. Bortezomib and ixazomib increased activated caspase 3 in HIV-infected J-Lat 10.6 cells in a dose- and time-dependent manner (*P* = 0.014 for 5 nM bortezomib; *P* = 0.004 for 10 nM bortezomib; *P* < 0.0001 for 50 nM and 100 nM ixazomib) (Fig. 4A and B). The same treatments only minimally increased activated caspase 3 expression in parental, uninfected Jurkat cells (Fig. 2H and 5D). In the J-Lat cells, bortezomib and ixazomib induced intracellular p24 expression (Fig. 4C, top), cleavage of caspase 3 (Fig. 4C, middle), and cleavage of PARP (Fig. 4C, bottom), consistent with these treated cells undergoing apoptosis.

In order to determine if the increased apoptosis in HIV-infected cells induced by ixazomib was dependent on Casp8p41 expression, we utilized JB-6 cells, which are procaspase 8 deficient and therefore resistant to HIV-induced apoptosis (15). Our strategy exploited our previous observation that procaspase 8-deficient JB-6 cells reconstituted with HIV protease-resistant procaspase 8 (Casp8RN) do not die following HIV infection, whereas JB-6 cells reconstituted with HIV protease-cleavable wild-type procaspase 8 (Casp8) do die from HIV infection (15). JB6 cells were infected with vesicular stomatitis virus (VSV)-G-pseudotyped HIV and 2 days later transfected with enhanced green fluorescent protein (EGFP)-Casp8, EGFP-Casp8RN, or EGFP empty -ector control (Fig. 4D and E). The cells were then treated with ixazomib or DMSO, and apoptosis (active caspase 3) in infected (p24^+^) cells expressing GFP (a marker of procaspase 8) was measured. Ixazomib increased active caspase 3 expression in HIV-infected (p24^+^, -bright, and -dim) cells expressing wild-type procaspase 8 (*P* = 0.005) (Fig. 4F), but not in HIV-infected cells expressing the mutant, noncleavable procaspase 8 or in cells expressing GFP alone. The same effect was noted when the analysis was restricted to p24-bright cells only (Fig. 4G, active caspase 3 staining) (mean fluorescence intensity [MFI] of 564 versus 385, comparing ixazomib- versus control-treated wild-type [WT]-Casp8-positive cells, respectively, as opposed to active caspase 3 staining [MFI of 285 in both ixazomib- and control-treated Casp8RN-expressing cells]) in order to decrease the possibility that the p24-positive cells were false positive. These data indicate that the increase in apoptosis induced by ixazomib is dependent upon expression of procaspase 8 and the ability of procaspase 8 to be cleaved by HIV protease.

### Proteasome inhibitor treatment kills infected cells more than uninfected cells in coculture.

We next evaluated the effects of bortezomib and ixazomib on HIV-infected J-Lat 10.6 cells compared to the parental non-HIV-infected Jurkat cells using a clonotypic assay in which we evaluated cell survival within mixed populations.

First, we confirmed that HIV-infected J-Lat 10.6 cells and uninfected Jurkat T cells have similar propensities to undergo apoptosis in response to non-HIV apoptotic stimuli (CH-11, a Fas agonist, and camptothecin, a topoisomerase inhibitor) (Fig. 5A and B). Since J-Lat 10.6 and Jurkat cells are phenotypically indistinguishable, we labeled the chronically HIV-infected J-Lat cells with the lipophilic dye CellTracker Orange (CTO) and mixed the cells 50:50 with uninfected, unlabeled Jurkat T cells. These cell mixtures were then treated with vehicle control, bortezomib, or ixazomib. Conceptually, if the treatments cause similar degrees of killing in uninfected J-Lat 10.6 cells and in Jurkat cells, then the proportion of labeled CTO^+^ J-Lat cells should remain at ∼50% over time. Bortezomib and ixazomib treatments both resulted in a decrease in the proportion of viable CTO-positive J-Lat 10.6 cells (Fig. 5C) (*P* < 0.001). Flow cytometric analysis demonstrated that, despite low-level caspase 3 induction in Jurkat cells, there were significantly more active caspase 3-positive HIV-infected J-Lat cells than uninfected Jurkat cells under the same culture conditions (*P* < 0.01 for 10 nM bortezomib and 100 nM ixazomib) (Fig. 5D and E).

We have previously shown in primary CD4^+^ T cells from ART-suppressed patients that reactivation from latency induces Casp8p41 expression (15). Therefore, we postulated that proteasome inhibitor treatment of cells from ART-suppressed HIV-infected donors might (i) reactivate HIV, (ii) stabilize Casp8p41, and (iii) result in the death of more HIV-infected cells than uninfected cells. Since clinical experience indicates that ixazomib does not cause significant leukopenia (35, 36) whereas bortezomib is associated with significant gastrointestinal and hematological toxicity (37), we used ixazomib for these studies. To first assess the clinical relevance of our findings, we infected activated primary CD4 T cells with a clinical dual-tropic HIV-1 isolate (HIV-1_RF_), treated with ixazomib or control, and assessed for caspase 3 activation over time (Fig. 5F). Ixazomib increased active caspase 3 expression in HIV-1_RF_-infected and -exposed cells (*P* < 0.0001), but not in mock-infected cells. This is consistent with our findings in laboratory-adapted HIV-1 strains (compare Fig. 2E and H) and suggests that ixazomib's effect may be clinically relevant.

Given the very low frequency of HIV-infected cells in blood circulation (38), it is not possible to monitor the death of latently infected cells compared to uninfected cells; we therefore analyzed the HIV DNA levels before and after treatment in patient samples. CD4 T cells from 11 HIV-positive ART-suppressed patients were treated with ixazomib or control, and the HIV DNA content was measured by assessing total cell-associated HIV DNA content with digital-droplet PCR (ddPCR) (39). Ixazomib treatment reduced total cell-associated HIV DNA by a median of 35% (interquartile range [IQR], 7% to 42%; *P* = 0.007) (Fig. 5G, top). To independently verify this result, we treated cells from an additional 8 patients with ixazomib, and an independent laboratory, blinded to sample identification, analyzed the integrated HIV DNA content using a previously validated assay (40, 41). Ixazomib treatment also reduced integrated HIV DNA in the second group (median reduction in integrated HIV DNA, 69% [IQR, 58 to 89%]; *P* < 0.001) (Fig. 5G, bottom).

## DISCUSSION

There is increasing appreciation that the biological processes that favor survival of cancer cells also operate in cells containing the transcriptionally silent reservoir for HIV; examples are epigenetic modifications of DNA and enhanced expression of checkpoint inhibitors (42, 43). Following the initial description of the HIV protease Casp8p41 pathway of HIV-infected-cell killing (13), the simultaneous advances in understanding of the control of proapoptotic Bak (44) allowed mechanistic insights into how the catalytically inert Casp8p41 protein might bind and activate Bak to initiate cell death. Indeed, we now know that Casp8p41 adopts an alpha-helical conformation that functions as a BH3-like domain that binds with nanomolar affinity to the BH3 groove in Bak (21), causing the death of HIV-infected cells. As other Bcl2 family members contain BH3 grooves and therefore can bind proteins containing BH3 domains (45), our finding that Casp8p41 binds Bcl2 and Bak with similar affinities (22) provides a mechanistic basis for how cells that generate Casp8p41 might escape the prodeath effects of Casp8p41. Indeed, selectively occupying the BH3 groove in Bcl2 with venetoclax allows Casp8p41 to interact with Bak, and infected-cell death is favored (23). In the current report, we extend this understanding to show that the Casp8p41-Bcl2 complex is polyubiquitinated and targeted to the proteasome for degradation. Blocking this pathway with ixazomib or bortezomib allows Casp8p41 to accumulate, thereby reversing the Bcl2 restriction of HIV-infected-T cell death. Also of additional benefit is the previously unreported finding that ixazomib independently drives HIV reactivation, which is, of course, a necessary step in generating Casp8p41 production. At present, it is unknown if Casp8p41 is expressed in HIV-infected macrophages or if proteasome inhibitor treatment of infected macrophages would have the same effect as in T cells. This will be important to study, as macrophages are naturally resistant to HIV-induced cell death and contribute to the HIV-1 reservoir (46).

**FIG 6:**
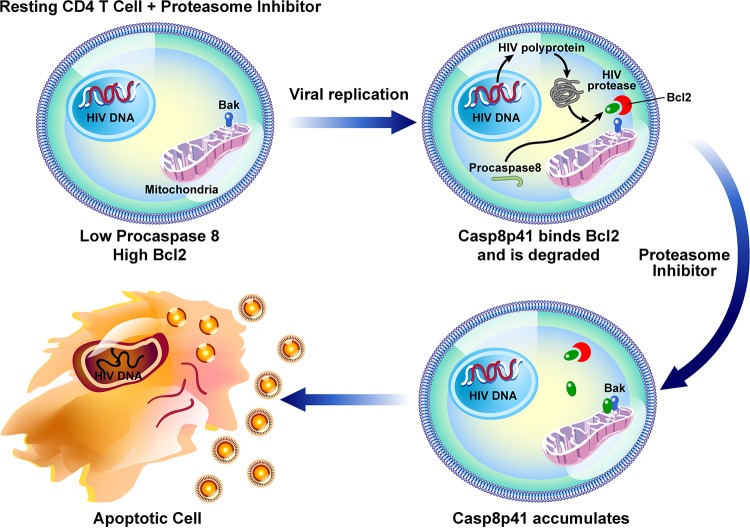
Schematic model of the effects of proteasome inhibitors of HIV-infected cells. Proteasome inhibitors directly induce viral replication, which generates Casp8p41, which is bound and inhibited by Bcl2 and then targeted to the proteasome for degradation. Proteasome inhibitor-mediated blockade of Casp8p41 degradation favors accumulation of Casp8p41, which overcomes Bcl2 restriction, causing death of the reactivated, infected cell. Because Casp8p41 is not present in uninfected cells, this pathway does not operate in uninfected bystander cells.

Our data demonstrate that an oral proteasome inhibitor that is clinically dosed once weekly functions dually to reactivate HIV and to increase Casp8p41 expression, thereby inducing apoptotic death of T cells in which HIV replicates (Fig. 6). Killing is preferential for HIV-infected cells because cell death is dependent in part upon the presence of functional Casp8p41 (Fig. 4F), which is produced only by HIV protease (13, 14). It should be noted that cell death is also increased to some degree in ixazomib-treated, GFP-negative Jurkat T cells exposed to HIV-GFP (Fig. 2J), as well as ixazomib-treated, GFP-negative J-Lat cells (Fig. 4A). However, we have previously shown that GFP expression alone does not differentiate infected versus uninfected dying cells, as GFP expression is lost during apoptosis (22). Also, these GFP-negative cells were exposed to HIV; therefore, individual cells, even if uninfected, could be exposed to other death-inducing stimuli, such as FasL, HIV gp120, or HIV Vpr. Therefore, the important experimental controls used were uninfected, unexposed cells.

This preferential, proapoptotic effect of ixazomib causes two surrogate measures of HIV reservoir size (total cell-associated and integrated HIV DNA levels) to decrease following single-dose treatment *ex vivo*. Acknowledging that the full clinical significance of *ex vivo* HIV DNA content reductions is unknown, and given that a significant fraction of HIV DNA is replication or otherwise defective (47), additional studies investigating the impact of multidose ixazomib on HIV dynamics using large-volume leukophoresis samples are ongoing via a pilot clinical trial of ixazomib in ART-suppressed HIV-positive patients (ClinicalTrials.gov identifier NCT02946047).

## MATERIALS AND METHODS

### Cells.

All HIV-positive donors were on antiretroviral therapy and had a plasma HIV RNA load of <48 copies/ml. Where indicated, peripheral primary CD4 T cells were purified by negative selection, using RosetteSep human CD4^+^ T cell enrichment cocktail (Stemcell Technologies) or an EasySep human CD4^+^ T cell isolation kit (Stemcell Technologies); we routinely achieve >95% CD4 T cell purity by these techniques. The following cell lines and sources were used: Jurkat T cells (Jurkat e6.1; ATCC; catalog number TIB-152), 293T cells (HEK-293T; ATCC; catalog number CRL-3216), J-Lat 10.6 cells (NIH AIDS Reagent Program), and JB-6 cells (provided by S. Nagata [48]).

### Antibodies.

The following antibodies were used for immunoblotting or immunoprecipitation: anti-HA peroxidase (3F10) rat monoclonal antibody (catalog number 12013819001; Roche, Indianapolis, IN), mouse anti-caspase 8 antibody (received from Marcus E. Peter, Northwestern University, Chicago, IL), mouse monoclonal anti-ubiquitin (P4D1) antibody (sc-8017; Santa Cruz), anti-Bcl-2 (C21) rabbit polyclonal IgG (catalog number sc-783; Santa Cruz), monoclonal anti-HIV p24 (catalog number 530; NIH AIDS Reagent Program), anti-GAPDH (glyceraldehyde-3-phosphate dehydrogenase) (14C10) rabbit MAb (catalog number 2118; Cell Signaling), histone deacetylase (HDAC) antibody sampler kit (catalog number 9928; Cell Signaling), anti-phospho-NF-κB-p65 (Ser 536) rabbit MAb (catalog number 3033; Cell Signaling), anti-NF-κBp65 (D14E12) rabbit MAb (catalog number 8242; Cell Signaling), anti-phospho-IκBα (Ser32) (14D4) rabbit MAb (catalog number 2859; Cell Signaling), anti-IκBα (L35A5) mouse MAb (amino-terminal antigen; catalog number 4814; Cell Signaling), anti-cleaved caspase 3 (Asp175) antibody (catalog number 9661; Cell Signaling), and mouse anti-human PARP (catalog number 556494; BD Pharmingen). The following antibodies were used for flow cytometry: phycoerythrin (PE)-conjugated mouse anti-HIV core antibody (Beckman Coulter) and allophycocyanin (APC)-conjugated rabbit anti-active caspase 3 antibody (BD Pharmingen; 1:100).

### DNA and viral constructs.

The following reagents were obtained through the NIH AIDS Reagent Program, Division of AIDS, NIAID, NIH: HIV-1_IIIB_, from Robert Gallo, and HIV-1_RF_, from Dean Winslow. The envelope-defective proviral GFP-HIV-Nef-Vpr 426-bp plasmid used for Fig. 2F to H has been previously described (21). The HIV-1 envelope-defective proviral plasmid HxBRUR^−^/Env^−^ was obtained from É. Cohen (University of Montreal, Montreal, Canada) (49). Individual proviral constructs were transfected into 293T cells, along with VSV-G expressor (SVCMV-VSV-G); 48 h later, culture supernatants were collected, clarified, and ultracentrifuged to pellet pseudotyped virus. Plasmids pEGFPC1, pEGFP-procaspase 8 Wt, and pEGFP-procaspase 8 RN (caspase 8 that is resistant to HIV protease cleavage due to mutation of Arg [R] 355, Asn [N] 356, Phe [F] 355, and Phe [F] 356) have been previously described (20).

### Flow cytometry.

Jurkat T cells were transfected with GFP-empty vector and GFP-Casp8p41 constructs, as previously described (21). Intracellular expression of active caspase 3, HIV p24 antigen, and Casp8p41 were assessed by flow cytometry, as previously described, using appropriate isotype controls for specific determination of expression (17, 22). For the clonotypic assay, J-Lat 10.6 cells were stained with CellTracker Orange (Molecular Probes) according to the manufacturer's protocol. The labeled J-Lat 10.6 cells were washed and cocultured with unlabeled Jurkat cells at a ratio of 1:1. The cocultured cells were treated with DMSO, bortezomib, or ixazomib at the indicated concentrations for 3 days before being washed and fixed with 2% paraformaldehyde for 24 h prior to analysis. For the experiments shown in Fig. 4D to F, the following gating strategy was used. GFP-positive cells were determined using untransfected controls and used for downstream analyses. HIV-1 p24- and active caspase 3-positive cells were determined using isotype controls. HIV-1 p24-bright cells were identified on the flow plot as those cells which were present between 10^4^ and 10^5^ on the relevant axis by visual cloud gating. For the analysis shown in Fig. 4F, the calculation performed was as follows: (percentage in quadrant 2)/(percentage in quadrant 2 + percentage in quadrant 3) × 100. Fluorescence-activated cell sorting (FACS) analysis was performed on either a FACScan or Fortessa flow cytometer (BD Biosciences) as needed, based on multicolor parameters. Data analysis was performed using FlowJo software (Tree Star, Inc.).

### IncuCyte analysis.

Jurkat cells were infected at a multiplicity of infection (MOI) of ∼1.0 with VSV-G GFP HIV in the presence of 6 μg/ml Polybrene (Sigma-Aldrich) for 6 h, washed, and treated or not with ixazomib (100 nM). Cell death was measured with a real-time imaging IncuCyte system (Essen BioScience) using the cell permeability dye IncuCyte cytotox red reagent (catalog number 4632; Essen BioScience). Analysis of the data was done using IncuCyte Zoom software (2016B).

### Analysis of ubiquitination.

Casp8p41 protein was analyzed for ubiquitin linkage specificity with a UbiCrest deubiquitinase (DUB) enzyme set (catalog number K-400; Boston Biochem, Inc.) (50). 293T cells transfected with Casp8p41-HA were lysed and immunoprecipitated using an anti-HA affinity matrix (Roche), and Casp8p41 bound to the HA matrix was mixed with 1× DUB reaction buffer with 1× concentrations of enzymes OTUB1, GST_AMSH, OTUD3, His_6_-TRABID, CEZANNE, YOD1, OUTLIN, and USP2 and incubated at 37°C for 30 min. Casp8p41-HA protein was eluted and resolved by SDS-PAGE and immunoblotted with HA-horseradish peroxidase (HRP) (Roche).

### HIV-LTR-Luc and HIV-LTR ΔKB-Luc reporter constructs.

HIV-1 luciferase-expressing HIV-1 coexpressing Renilla reniformis (pNL-LucR) was obtained from C. Ochsenbauer (University of Alabama at Birmingham, Birmingham, AL), as previously reported (51). The pNL-LucR stocks were prepared by Lipofectamine LTX transfection of 293T cells, and 60 to 72 h posttransfection, virus was concentrated from culture supernatants by ultracentrifugation, followed by determination of the p24 concentration. All the viral aliquots used for infection underwent one freeze-thaw cycle. Primary CD4^+^ T cells were HIV infected after activation using phytohemagglutinin (2 μg/ml) and interleukin-2 (IL-2) (50 IU/ml) for 48 h; then, ∼100 ng of p24 in 0.5 ml was added to 10^6^ cells for 6 h and then washed and cultured with 10 IU/ml IL-2. When indicated, cells were lysed with 1× Renilla luciferase lysis buffer and analyzed for Renilla luciferase activity. Viability was assessed using a CellTiter-Glo luminescent cell viability assay (Promega).

### Total cell-associated HIV DNA measurement and integrated HIV DNA measurement. (i) Quantification of HIV-1 DNA using Raindance digital droplet PCR.

DNA for analysis was extracted using a Qiagen DNA blood minikit (Qiagen, CA), linearized using a Covaris M220 focused ultrasonicator with 3.0 kb minitube blue (Covaris, MA), and quantified using a NanoDrop 2000 spectrophotometer. The fragmentation length was confirmed via gel electrophoresis. Raindance digital PCR was performed using HIV primers and probes specific for HIV Pol (Hxb2 positions 2536 to 2662) and an RPP30 (RNase P) primer-probe set as previously reported (39). PCRs were prepared by mixing 1 μg of sonicated genomic DNA, 900 nM primers, and 250 nM probe mixture with 25 μl of TaqMan genotyping master mix (Applied Biosystem, CA) and 2.5 μl of droplet stabilizer in 50 μl. PCR was conducted in a thermocycler with a heated lid (Genetouch thermal cycler; Bioer, Tokyo, Japan) with the following settings: 2 min at 50°C and a denaturation step of at 95°C for 10 min, followed by 45 cycles of 95°C for 15 s and 60°C for 1 min (using a 0.6°C/min ramp rate). Thermal-cycled samples were loaded onto the Raindrop Sense instrument, which uses a 488-nm laser to read the 6-carboxyfluorescein (FAM) and VIC fluorescence intensities per droplet. Data from each sample were converted to a 2-dimensional scatterplot displaying FAM intensity on the *x* axis and VIC intensity on the *y* axis. Gates used to count the droplet events with specific fluorescence properties were defined using graphical tools to outline regions from the positive control, and these gates were applied to all the samples. The positive droplet events were counted within each gate for each sample. The droplet counts were normalized to the RPP30 counts. Total HIV DNA was calculated and expressed in 1 million CD4 cells by the estimated number of RPP30 copies [(Pol/RPP30) × 2,000,000]. We validated the use of ddPCR for total HIV DNA estimation using 10 independent evaluations of samples containing a fixed ratio of uninfected Jurkat T cells and chronically HIV-infected 8E5 T cells. The coefficient of variance of the ddPCR was 3.8%, and the detection limit of the assay was found to be 45 HIV DNA copies per million cells.

### (ii) Integrated HIV DNA quantification.

DNA was isolated using a Gentra Puregene cell kit (Qiagen). Levels of integrated HIV DNA were measured by Alu-Gag quantitative PCR (qPCR), as previously described (40). Briefly, a first-step PCR mixture containing a forward primer specific for the human Alu element and a reverse primer specific for the HIV *gag* gene was followed by a nested real-time PCR with primers and a probe specific for the HIV LTR. The numbers of cells assayed per well ranged from 1,813 to 29,000. The number of cells assayed per well was based on total HIV levels to result in the tightest confidence intervals for binomial distribution (we targeted 30 to 80% positive wells at two dilutions) (40).

### Statistics.

Results are depicted as means and standard deviations (SD) unless specifically noted. The mean values of the results of independent experiments were compared using *t* tests, analysis of variance (ANOVA), or the nonparametric equivalent, as appropriate. The mean values of matched samples were compared using paired *t* tests. Time course experiments were compared using linear regression. All tests were two sided, and *P* values of less than 0.05 were considered statistically significant. Statistical analysis was performed using GraphPad Prism 6.

### Study approval.

Primary human peripheral blood mononuclear cells were obtained following informed consent according to Mayo Clinic Institutional Review Board (IRB)-approved protocols (IRB numbers 1036-03 and 13-005646) and a Hennepin County Medical Center Human Subjects Research Committee-approved protocol (HSR number 15-3934).
